# Parameter Optimization of Biodegradable Composite PLA–Wood with New-Generation Infill Pattern

**DOI:** 10.3390/biomimetics11020106

**Published:** 2026-02-02

**Authors:** Mehmet Kivanc Turan, Altug Bakirci, Yusuf Alptekin Turkkan, Fatih Karpat

**Affiliations:** 1 Department of Mechanical Engineering, Bursa Uludag University, 16059 Bursa, Türkiye; 2 R&D Department, Ermetal Otomotiv ve Esya San. Tic. A.S., 16110 Bursa, Türkiye; altug.bakirci@ermetal.com; 3 Department of Electronics and Automation, Bursa Uludag University, 16850 Bursa, Türkiye

**Keywords:** additive manufacturing, ANOVA, fused filament fabrication, PLA–wood bio-composite, Taguchi

## Abstract

The increasing interest in sustainable materials has led to the development of bio-based composites for additive manufacturing applications. This study aimed to investigate the influence of key printing parameters and new-generation infill patterns together on the maximum compressive force of PLA–wood bio-composites produced by Material Extrusion. By optimizing this material, low-cost wood-like products can be produced. New-generation 3D infill patterns (octet, cubic-subdivision, and lightning which is a biomimetic infill pattern) infill densities, printing temperatures, and layer heights were selected as variables/factors, and the Taguchi method was applied for design of the experiment. The signal-to-noise ratio and Analysis of Variance were used to evaluate the statistical significance and contribution of each parameter to the mechanical response. The signal-to-noise ratio indicated that the optimal printing settings were as follows: printing temperature, 205 °C; infill density, 80%; infill pattern, octet; and layer height, 0.2 mm (7123.4 N). ANOVA results showed that infill density was the most significant factor affecting maximum compressive force at 60%, while infill pattern also exhibited a notable effect. According to these results, infill density and infill pattern are the most important factors for achieving high compressive strength. These findings suggest that optimizing infill architecture and density can improve the mechanical performance of PLA–wood composites, also they can offer assistive design guidelines for lightweight and eco-friendly components.

## 1. Introduction

Conventional manufacturing methods are costly and impractical in some situations [1]. Prototype production, specialized design production, and limited volume production incur high costs in traditional manufacturing. For this reason, manufacturers have been focusing on finding alternative manufacturing techniques. Additive manufacturing is one of the ideal solution methods for low-volume production or special edition design [1]. For this reason, various sectors utilize additive manufacturing techniques [1,2,3,4,5,6,7,8,9]. Additive manufacturing can be described as creating a product layer by layer. Although there are many additive manufacturing types, such as Selective Laser Melting (SLM), Powder Bed Fusion (PBF), Stereolithography (SLA), and Direct Energy Deposition (DED) [1,2,10,11,12,13], probably the most used additive manufacturing type is Material Extrusion (MEX) [10,12]. Since MEX has a wide range of devices, from handmade to highly professional, in other words, MEX can be used at home and in a factory.

Many different materials can be used in MEX, such as Acrylonitrile Butadiene Styrene (ABS), Polyethylene Terephthalate Glycol (PET-G), Thermoplastic Polyurethane (TPU), and Polylactic Acid (PLA) [12,14,15]. PLA is especially widely used in the MEX additive manufacturing technique. Since PLA filament is a low-cost and environmentally friendly material, it can be recycled [16]. Sometimes it can be reinforced with some materials, for instance, wood, ceramic, polyhydroxyalkanoate, etc., for mechanical or visual expectations [10,11,15,16,17,18,19,20].

Printing parameter settings are very critical for MEX manufacturing. The production will probably meet mechanical or visual expectations if the user cannot use the optimal parameters set. There are many essential parameters for MEX, such as printing speed, raster angle, raster orientation, printing temperature, heated-bed temperature, infill density, and infill pattern [1,9,11,15,18]. When the literature was reviewed, it was seen that there are many parameter optimization studies about MEX manufacturing.

Turan et al. [1] investigated the effect of additive manufacturing printing parameters on the mechanical properties of the tensile sample. They used printing speed, raster angle, printing temperature, heated-bed temperature, and layer height as research parameters. tensile tests, shore D hardness tests, and roughness tests were used to evaluate mechanical properties in the study. As a result of the study, they observed that the most effective parameters for tensile strength were raster angle; for roughness, layer height; and for hardness, printing speed. Mahesh et al. [9] examined printing parameter effects on the tensile strength of PLA and PET-G tensile test samples. They used printing speed, layer height, infill density, and infill pattern. They observed that the infill pattern was the most effective parameter for PET-G, and layer height was the most effective parameter for PLA. Yermurat et al. [21] discussed combining ABS and PLA materials. They used infill pattern, infill density, and layer height as printing parameters. As a result of the study, they observed that the maximum tensile strength was achieved with a 0.3 mm layer height, 90% infill density, and a triangle infill pattern for a combined structure. They also compared PLA and ABS tensile samples produced with these parameters with this sample. They saw that the combined structure was stronger than ABS and PLA. Roj et al. [22] compared the parts produced by MEX and the parts produced by molding. They emphasized that tensile strength is associated with the density of the material. Tunçel et al. used a ceramic composite filament in their study. Infill pattern, infill density, nozzle diameter, wall line count, and layer height were chosen as factors for investigation and the Taguchi method. As a result of the study, they found that the most effective parameter for tensile strength was nozzle diameter. Additionally, increasing infill density and layer height also increased tensile strength.

Wood-reinforced PLA has recently become a research topic of great interest [20], because wood-reinforced PLA composites may be the most biodegradable and environmentally friendly type of PLA composite. Estakhrianhaghighi et al. [10] added wood to PLA. As a result of the study, it was observed that the maximum ultimate tensile strength was achieved by adding 2.5% wood (+9%). In addition, as the percentage of wood increased, the ultimate tensile strength values decreased. For instance, the maximum tensile strength has decreased by 13% for 85% PLA and 15% wood. Kianifar et al. [18] investigated the fatigue life of PLA–wood. They used a fully reversed bending test to determine the fatigue life of PLA–wood. For this purpose, four stress levels were selected: 7.5 MPa, 10 MPa, 12.5 MPa, and 15 MPa. As a result of the study, they emphasized a notable decrease in fatigue life between 7.5 MPa and 15 MPa as the stress level increased. Siddiqui et al. [17] compared pure PLA and PLA–wood in terms of their mechanical and flammability properties. Printing orientation was a research parameter for the mechanical test. They observed that pure PLA, which was produced with Y orientation, had the best tensile and flexural strength. In addition, the PLA–wood burning rate (52.81 mm/min) was higher than that of pure PLA (44.64 mm/min). Pathinettampadian et al. [16] investigated the effects of raster angle on the mechanical properties of PLA, PLA–wood, and a Multi-Layered Material (wood and PLA). Tensile, compression, and flexural tests were used to determine mechanical properties. PLA–wood had the worst mechanical properties in all tests and all raster angles. Chaidas and Kechagias [15] discussed the effects of layer height and printing temperature on the dimensional accuracy and surface quality of printed parts made from PLA–wood material. They observed that layer height was a more effective printing parameter in general. For instance, the layer height contribution ratio for average roughness (Ra) was 91.6%. Kechagias et al. [23] investigated the mechanical properties of PLA/coconut wood composite. They observed that maximum tensile strength increased with increasing printing temperature and layer height. Ulkir et al. [24] discussed the effects of printing parameters on the tensile and compression strength. They observed that tensile and compression strength increased with increasing infill density and decreasing layer height. However, continuous increases in the printing temperature continuously increase the tensile strength, while the compressive strength increases up to 200 °C and then decreases with further increases. Samykano et al. [25] researched the compression properties of PLA/coconut wood composite. They saw that compression strength increased with increasing infill density.

In additive manufacturing, tensile strength is generally the focus. However, materials are often subjected to different loadings, and examining these properties is also essential. Compressive strength is the most important of these. Kamer and Dogan [13] investigated the effects of infill pattern and compression axis on the compression performance of the PLA cubic part. They selected cubic, quarter cubic, grid, gyroid, and octet as infill patterns. They observed that the octet is the optimal infill pattern in general. Dave et al. [26] researched the effects of layer height, printing speed, and infill density on the compression strength of the PLA scaffold. They used signal-to-noise ratio (SNR) and Analysis of Variance (ANOVA) to analyze experiments. They found that the ideal printing parameters were a layer height of 0.2 mm, an infill density of 80%, and a printing speed of 40 mm/min. In addition, they observed that infill density was the most effective printing parameter for compression strength. Tunçel and Bayraklilar [27] investigated the effects of printing speed, wall thickness, and infill pattern on the compression strength. As a result of the study, they observed that the most effective parameter on the compression strength was the wall thickness. Additionally, they observed that the compression strength increased with increasing printing speed. Aydin [28] discussed wood-inspired cellular structure in his study. As a result of the compression tests, the author emphasized that using a 0.2 mm layer height provides cost and time savings compared to a 0.1 mm layer height.

Today, improving mechanical performance is an important research topic in additive manufacturing. Increasing mechanical strength without significantly increasing mass or reducing it is a fundamental task for designers and analysts. There are two main ways to achieve this: improving the material or improving the design. Improving the material, i.e., using different materials or strengthening it by creating composites with reinforced materials, meets the mechanical requirements. At this point, biologically reinforced elements can be used, especially in the development of composite materials. However, changing the material will increase costs. Simply using reinforced materials, such as biological structures (e.g., stone fill), may not pose a significant cost issue. For this reason, improving the design is becoming more prominent. In this regard, biomimetic designs have become an interesting research topic.

Many structures or elements in nature have attracted researchers due to their mechanical properties, prompting them to consider incorporating these into their designs or research. Bio-based or biomimetic structures are among today’s research topics. This is because many living and non-living entities in nature have unique designs that confer advantages, prompting scientists to study these topics [29,30,31,32,33,34,35,36]. For example, honeycomb structures are highly resistant to forces acting perpendicular to the comb. Another well-known example is ant nests. These nests appear to be designed with remarkable urban planning and also possess very high strength. Similarly, there are studies inspired by nacre or the shells of turtles. This is because they exhibit very high properties in terms of strength and durability. These and similar examples prompt scientists to draw inspiration from natural structures and incorporate them into their own research topics or hypotheses.

Sometimes PLA material is also combined with metal materials. The main reason for this is that PLA material is environmentally friendly and bio-based [37,38].

This study investigated the optimization of printing parameters and their effects on the compression properties of PLA–wood bio-composite filaments. This material is biodegradable, making it very important for a sustainable environment. The Taguchi method and Factorial ANOVA (General Linear Model) were used for these purposes. Minitab software version 17 was used for these processes. The study was conducted experimentally, contributing to the literature using by PLA–wood bio-composite filament because there are not enough publications for this composite material in the literature. There is no parameter optimization study focusing specifically on biomimetic 3D infill patterns. The use of the signal-to-noise ratio to detect the optimum parameter set, the application of ANOVA to determine the contribution ratios of printing parameters, and the selection of new-generation (3D) infill patterns (octet, lightning (bio-inspired), and cubic-subdivision), in particular, make this study even more significant because, when the literature was investigated, many studies investigated or used conventional (2D) infill patterns, such as concentric, grid, line, zig-zag, etc. In addition, the new-generation 3D lighting pattern is bio-inspired and stands out with its lightning flash, hairy root, and fractal branch biomimetic. This pattern is generally designed to support the top layer. In addition, other infill patterns, octet, cubic-subdivision, and concentric, can also be considered bio-inspired/biomimetic infill patterns. The cubic-subdivision infill pattern resembles cancellous bone structure, the concentric infill pattern is biomimetic of tree rings, and the octet infill pattern can be seen as the internal structure of face-centered cubic materials. This study will be a significant contribution to the literature, as it presents an article supported by optimization and statistical analysis, featuring 3D infill patterns (bio-inspired/biomimetic infill patterns) on PLA–wood.

## 2. Materials and Methods

### 2.1. Materials

**Figure 1:**
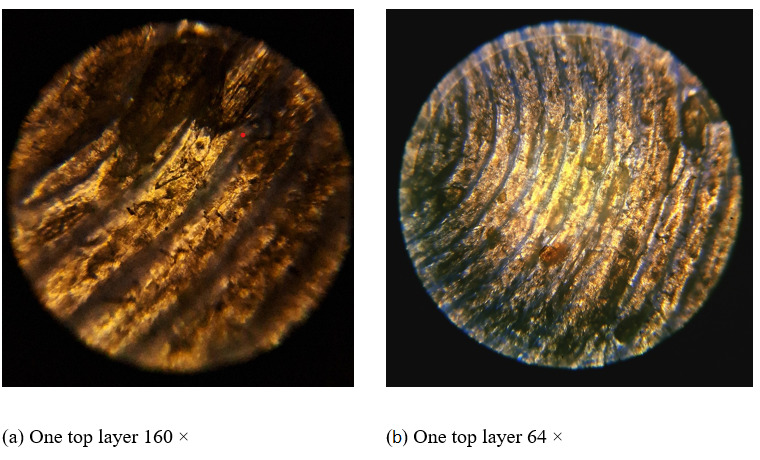
Microscope image of a specimen printed from PLA–wood composite filament.

This study utilized 1.75 mm diameter PLA–wood bio-composite filament (Figure 1) as the material, which contained 30% wood (Porima brand, made in Yalova/Türkiye, with no information provided about the type of wood used).

The filament manufacturer recommends a printing temperature range of 200–230 °C and a heated-bed temperature range of 0–60 °C. Both PLA and wood are not only biodegradable but also recyclable. For this reason, the usage rate of PLA–wood bio-composite filament is expected to increase. The filament was used as it was removed from the vacuum-sealed package.

### 2.2. Parameter Selection

**Figure 2:**
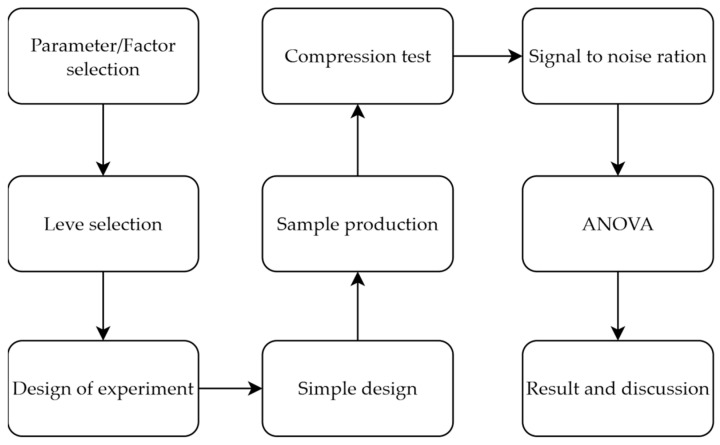
Experimental schematic workflow of this study.

As the introduction emphasizes, selecting ideal parameters is critical for additive manufacturing. Printing parameters are very effective in terms of mechanical properties, visual properties, etc. Printing temperature, infill pattern, infill density, and layer height were selected as investigation printing parameters. The main objective of this study is to optimize compression properties. The flowchart of this study is shown in Figure 2. Minitab 17 software was used for experimental design and statistical analyses, and the statistical significance value in ANOVA is a *p*-value ≤ 0.05.

The full factorial experiment design is very costly and time-consuming, making it difficult to detect the optimum parameter set. The Taguchi method saves costs and time by reducing the number of experiments and finding the optimum parameter set [1,39,40,41]. This method uses orthogonal arrays, and L16 orthogonal arrays are used in this study. This study used the Taguchi method with four factors and four levels, the following were used as research parameters: printing temperatures were 205 °C, 210 °C, 215 °C, and 220 °C (these values were selected according to the manufacturer’s recommended temperature range); infill patterns were lightning, concentric, cubic-subdivision, and octet; infill densities were 10%, 20%, 40%, and 80%; and layer heights were 0.1 mm, 0.15 mm, 0.2 mm, and 0.25 m. This parameter range has been selected based on the filament manufacturer’s recommendations and preliminary studies. Infill patterns were selected to resemble bio-based/biomimetic structures. It is generally recommended to use a maximum layer height of 75% of the nozzle diameter [19]. In addition, the manufacturer of the 3D printer used in this study recommends a minimum layer height of 0.1 mm [19]. For this reason, the L16 orthogonal array was selected. The experimental design is presented in Table 1.

**Figure 3:**
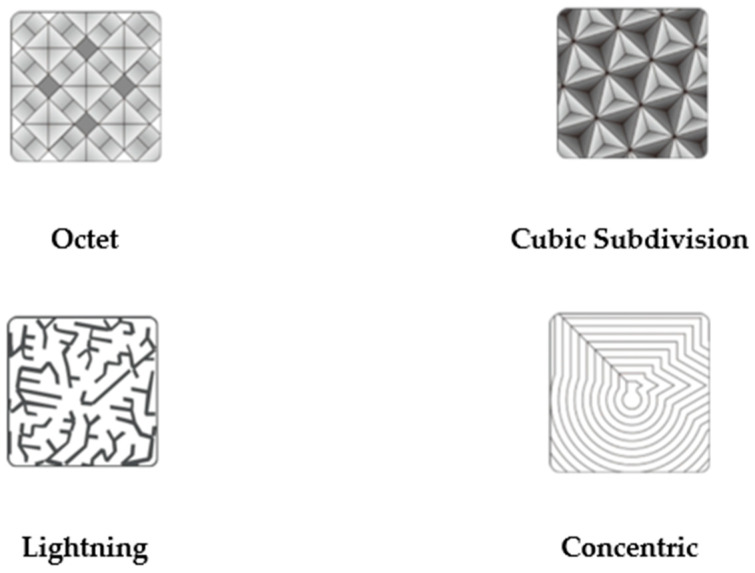
New-generation (3D) infill patterns (octet, cubic-subdivision, and lightning) and 2D infill pattern (concentric) (Shenzhen Creality 3D Technology Company (Shenzhen, China), 2023).

As emphasized in the introduction section, cubic-subdivision, octet, and lightning are new-generation (3D) infill patterns (Figure 3). These infill patterns are new-generation infill patterns that can generally be classified as three-dimensional infill patterns. These patterns were selected from the slicer program.

### 2.3. Manufacturing Process

**Figure 4:**
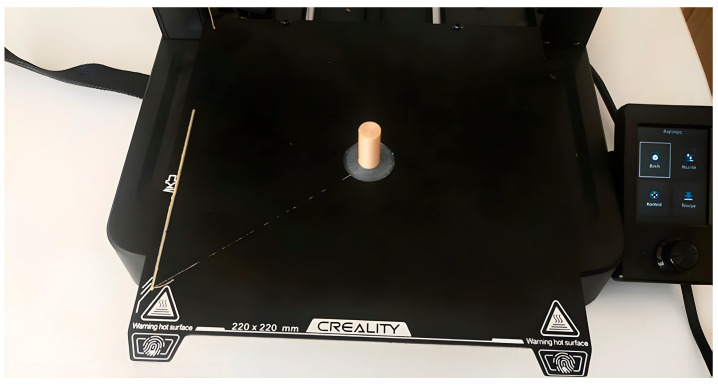
**Three-dimensional** printer and sample.

After that, the printing process was started under room conditions. Creality Print software was used as a slicer program. The Creality Ender 3 V3 SE 3D (made in Shenzhen, China) printer was used in the study; this device is shown in Figure 4. This device has a 220 × 220 × 250 mm printing volume and a 0.4 mm nozzle diameter. Table 2 gives the fixed parameters.

**Figure 5:**
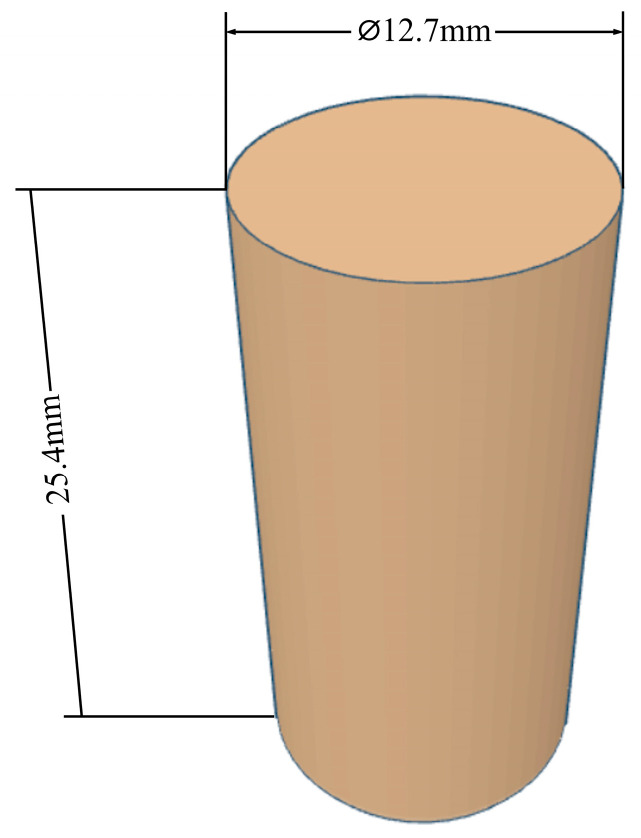
Compression test sample sizes.

Three samples were produced for each experiment set number, and ASTM D695 was used to design the sample. Figure 5 shows sample sizes.

### 2.4. Testing Process

**Figure 6:**
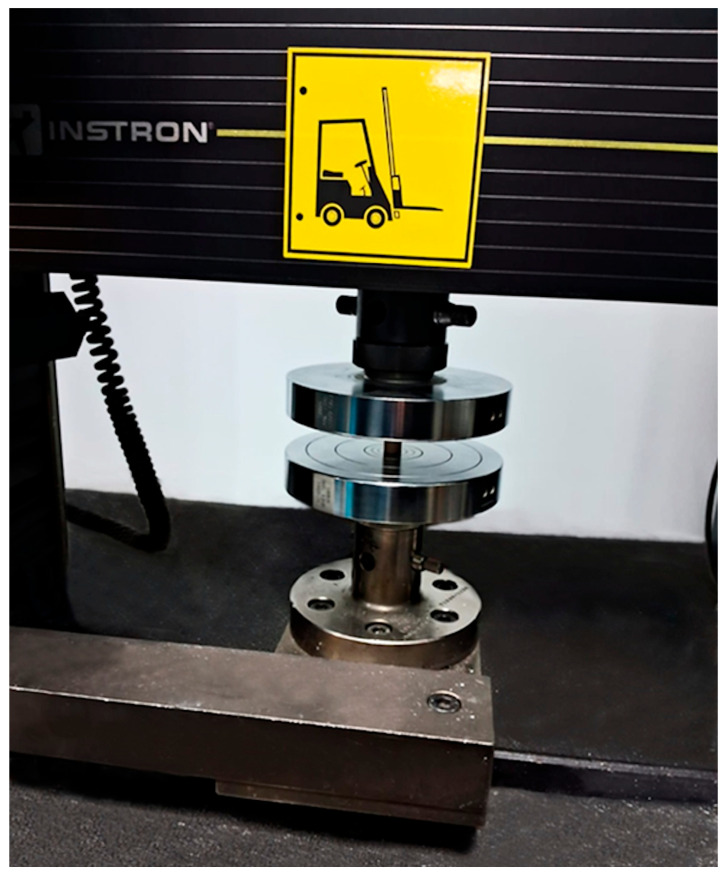
Compression test machine.

After the production process, the testing process began. An Instron 5582 (Norwood, MA, USA) test machine was used for the compression test at room conditions. The test machine is shown in Figure 6.

Compression tests were conducted up to a displacement of 12.7 mm at a test speed of 5 mm/min, and the maximum compression forces were recorded. The primary reason is that it was assumed that maximum compression force would be achieved when the sample shrunk to half its original size, and the same process was applied to all samples. This assumption is based on preliminary tests. After the test process, the results analysis processes began. At this point, signal-to-noise ratio and ANOVA were used. The Taguchi method utilizes a signal-to-noise ratio to evaluate results and identify the optimal parameter set. The signal-to-noise ratio (SNR), as calculated using the Taguchi method, has a more specific and powerful meaning than the standard signal-to-noise concept. This ratio measures not only how noisy our system is, but also how robust our design is against external factors (noise factors). The fundamental aim of Taguchi philosophy is to make process or product quality as insensitive as possible to changes in uncontrollable factors (noise). SNR summarizes this robustness in a single statistical value. There are three types of signal-to-noise ratios: larger is better, smaller is better, and nominal is the best. This study employed the ‘larger-is-better’ principle due to the maximum compression force desired (Equation (1)) [40,41].

(1) $$S/N=-10\log\left[\frac{1}{n}\sum_{i=1}^{n}\frac{1}{y_{i}^{2}}\right]$$

In Equation (1), *y_i_* represents the response data from the experiment for the i-th parameter and *n* denotes the experiment number.

Additionally, the contribution of printing parameters was determined using ANOVA. These calculations were realized with Minitab software.

## 3. Results and Discussion

### 3.1. Test Results

**Figure 7:**
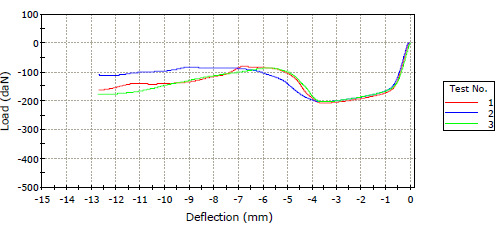
One of the force–displacement graphs obtained in the study.

**Figure 8:**
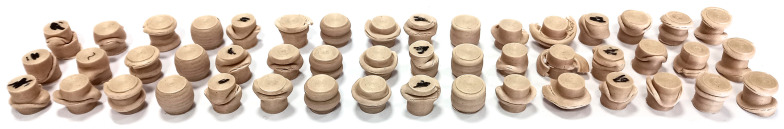
The compression samples are viewed at the end of the compression test.

First, the compression test results (Figure 7 and Figure 8) are presented in Table 3. The black dots (Figure 7) in the image are due to the support of the upper part of the sample in the lightning infill pattern. Therefore, markings have been made to ensure that the pressing is from the upper part of the sample. Deformation modes (Figure 7) are the same within a set, but they are usually different between sets. This is because each set contains different printing parameters. The averages of the maximum compression forces of each experimental set are shown in the table. For this purpose, the arithmetic mean of the three repetitions performed for each set was taken. The coefficients of variation were all below 4%, and in many cases, around 1%. According to the results, the fourth experimental set, printed at 205 °C with a layer height of 0.25 mm, an octet infill pattern, and an infill density of 80%, exhibited the strongest resistance to a compression force of 7123.4 N. The lighting pattern is noteworthy as a standard feature of the weakest samples, around 2000 N and lower. Despite an 80% infill density, the lighting infill pattern could withstand a compression force of 2025 N. The thirteenth experimental set, which had an 80% infill density and a lightning infill pattern, exhibited less resistance force than half the other samples with an 80% infill ratio (concentric infill pattern: 6977.3 N, cubic-subdivision infill pattern: 5191.6 N, octet infill pattern: 7123.4 N).

### 3.2. Taguchi Results and Discussions

**Figure 9:**
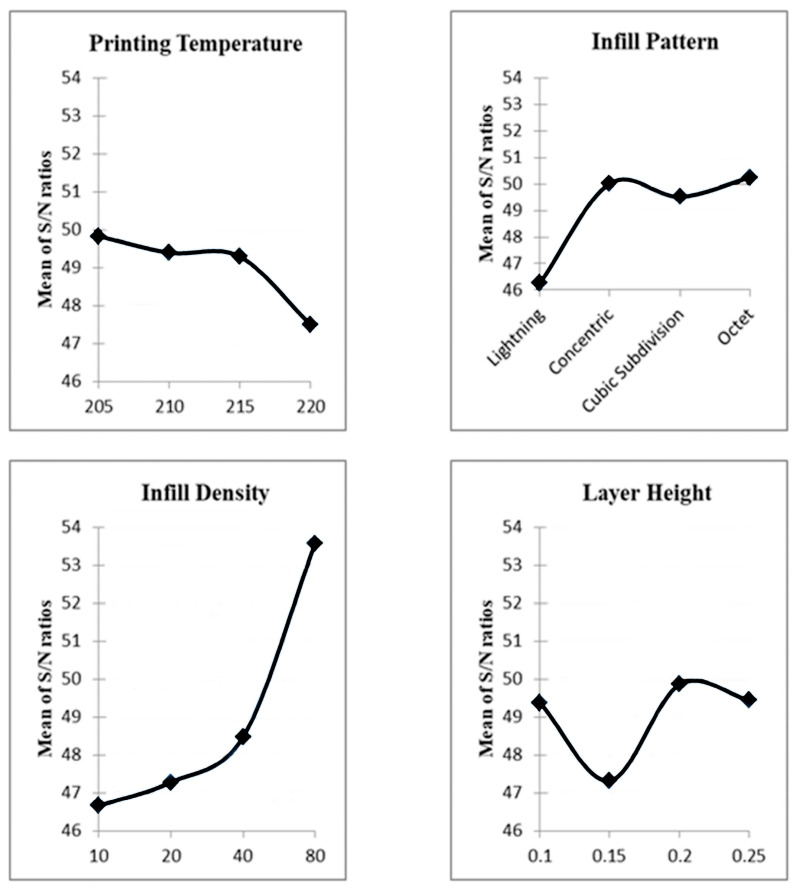
Taguchi signal-to-noise results.

To better understand the results, the Taguchi results need to be discussed. Taguchi graphics are shown in Figure 9, and the Taguchi result table is given in Table 4. Each level shows the signal-to-ratio result for a selected factor level. For example, layer heights correspond to the following levels: level 1, 0.1 mm; level 2, 0.15 mm; level 3, 0.2 mm; and level 4, 0.25 mm. The results corresponding to these factor levels are presented graphically in Figure 9. The Taguchi signal-to-noise ratio results yield the ideal parameter set, and the effect ranking of the parameters is also displayed.

**Figure 10:**
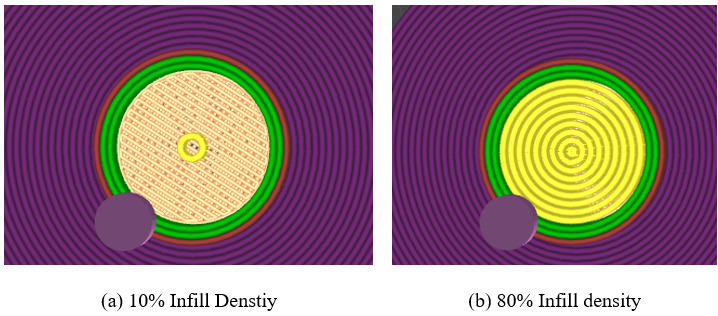
Infill structure depends on infill densities.

When Figure 9 and Table 4 were investigated, it was clearly seen that when the printing temperature increased, the maximum compression force decreased. The main reason for this situation can be that the filament contains wood parts, and wood parts can be burned with the increase in temperature [17,20]. Thus, blanks or pores occur, and the maximum compression force decreases. Additionally, each filament has an optimal printing temperature, which depends on its internal structure. These results are compatible with Ulkir et al.’s study [24]. On the other hand, when the infill pattern effects were examined, it was crystal clear that the lightning was the worst infill pattern. The leading cause of this result is that the infill pattern begins at a certain height. So, samples with this infill pattern are more porous and less durable. Although there is no significant difference between octet, concentric, and cubic-subdivision, octet was the high-strength infill pattern, which can be said that this result is compatible with the Kamer and Dogan study [13]. In addition, these results are consistent with the amounts of material used in parts produced with these infill patterns and the same parameters. Increasing the infill density increased the maximum compression force, as expected. As the infill density increases, the sample becomes fuller, and its strength increases (Figure 10—the yellow sections show the concentric infill pattern’s densities). This result is consistent with the literature [24,25,26]. While the ideal layer height appears to be 0.2 mm, this result is consistent with Dave et al.’s work [26].

The optimum parameters are set accordingly, since the larger-is-better principle is used in the signal-to-noise ratio, the maximum value in Figure 9 has been selected: printing temperature is 205 °C, infill density is 80%, infill pattern is the octet, and layer height is 0.2 mm. The fourth experiment was nearly identical to this set. Only the layer height differed (0.25 mm), but the signal-to-noise ratio results for the 0.2 mm and 0.25 mm layer heights were very close (Table 4, Figure 9), and maximum compression force was achieved in the fourth experiment. This result indicates that the outcomes are reliable.

### 3.3. ANOVA Results and Discussions

**Figure 11:**
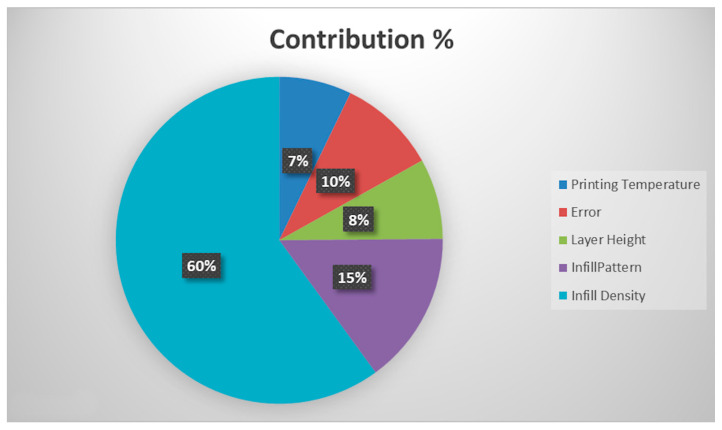
Contribution ratios of sources.

When Table 5 and Figure 11 were examined, the ANOVA and signal-to-noise ratio results were consistent. Infill density was the most effective parameter on maximum compression force, with 60.01%. The *p*-value of infill density was almost ≤0.05. It means that this hypothesis is statistically logical. However, using the Taguchi method to design experiments limited the data. This situation prevented the collection of the necessary experimental data. Since the Taguchi method reduces the number of experimental sets (Taguchi reduced the 256 experimental sets to 16), it cannot be determined exactly whether the parameters are statistically effective or not, and the two-factor interaction effects of the parameters cannot be observed. It was observed that the infill pattern (15.12%) and layer height (7.97%) were effective, respectively. In addition, according to this result, the printing temperature had a minimal effect on the maximum compression force of the samples. Error contribution was more significant than layer height and printing temperature in contributing to the ANOVA results. This result was due to the use of the Taguchi method for the design of experiments and its negative effects, as previously mentioned. However, due to limited data, the results of the binary interaction are inconclusive. These values are added to the error. This is clearly seen in the study by Kisin et al. [19].

## 4. Conclusions

Additive manufacturing is one of the most attractive research topics in the engineering area. Not only is research on additive manufacturing methods critical for this purpose, but material research is also critical for this purpose. Nowadays, detecting the effects of printing parameters and optimum/optimal printing parameter sets is one of the important research topics. At this point, MEX is the most used additive manufacturing technique. This study investigated the effects of layer height, printing temperature, infill density, and infill pattern for PLA–wood bio-composite filament. In particular, new-generation (3D) infill pattern types were used for the infill patterns. These infill patterns are not only 3D but also biomimetic. In addition, the concentric infill pattern is also biomimetic. The Taguchi method was used for the design of experiments. It was clearly seen that the most effective printing parameter was the infill density. There are two crucial conclusions:

• As a result of ANOVA, it was determined that infill density can affect the compression test results by 60%. High strength cannot be achieved with low infill density.
• In addition, the lightning infill pattern is not an appropriate infill pattern for maximum compressive force, because the infill pattern is designed only to support the upper section. In other words, its focus on providing resistance to the upper layer rather than the structure’s total load capacity results in a decrease in maximum compression force, indicating that this infill pattern cannot be used for overall structural strength.

The most challenging aspect of the study was the use of a 0.4 mm nozzle during the production phase. Using a larger nozzle diameter for reinforced composites would facilitate production. Statistically, returning to a full factorial experimental design by reducing the number of factors and levels would help to present the results more clearly and reveal interactions. The effect of changing the wood material, the type of PLA material addition (granules or fibers), the effect of particle size in granule additions, the effect of length and thickness measurements in fiber additions, the effect of printing parameters according to these, and the complex evaluation of these together will be important research topics for future studies. A comprehensive study will also be conducted on the effect of layer height, clearly demonstrating all its effects.

## Figures and Tables

**Table 1:** Design of experiment.

Experiment Set Number	Printing Temperature (°C)	Infill Pattern	Infill Density (%)	Layer Height (mm)
1	205	Lightning	10	0.1
2	205	Concentric	20	0.15
3	205	Cubic-Subdivision	40	0.2
4	205	Octet	80	0.25
5	210	Lightning	20	0.2
6	210	Concentric	10	0.25
7	210	Cubic-Subdivision	80	0.1
8	210	Octet	40	0.15
9	215	Lightning	40	0.25
10	215	Concentric	80	0.2
11	215	Cubic-Subdivision	10	0.15
12	215	Octet	20	0.1
13	220	Lightning	80	0.15
14	220	Concentric	40	0.1
15	220	Cubic-Subdivision	20	0.25
16	220	Octet	10	0.2

**Table 2:** Fixed printing parameters and sample properties.

Fan Speed Ratio (%)	Printing Speed (mm/s)	Heated-Bed Temperature (°C)	Nozzle Diameter (mm)	Sample Diameter (mm)	Sample Length (mm)
100	60	60	0.4	12. 7	25.4

**Table 3:** Compression test results.

Experiment Set Number	Printing Temperature (°C)	Infill Pattern	Infill Density (%)	Layer Height (mm)	Average Test Results (N)	CV (%)
1	205	Lightning	10	0.1	2052.3	1.15
2	205	Concentric	20	0.15	2205.7	0.49
3	205	Cubic-Subdivision	40	0.2	2875	3.70
4	205	Octet	80	0.25	7123.4	0.26
5	210	Lightning	20	0.2	2172.4	1.48
6	210	Concentric	10	0.25	2239	1.27
7	210	Cubic-Subdivision	80	0.1	5191.6	1.82
8	210	Octet	40	0.15	3007.8	2.28
9	215	Lightning	40	0.25	1965.6	1.33
10	215	Concentric	80	0.2	6977.3	0.84
11	215	Cubic-Subdivision	10	0.15	2174.5	0.74
12	215	Octet	20	0.1	2425.2	2.05
13	220	Lightning	80	0.15	2025	2.67
14	220	Concentric	40	0.1	2924.8	3.19
15	220	Cubic-Subdivision	20	0.25	2475.1	1.93
16	220	Octet	10	0.2	2162.4	0.37

**Table 4:** Taguchi signal-to-noise results table.

Factors	Printing Temperature	Infill Pattern	Infill Density	Layer Height
Level 1	49.84	46.25	46.67	49.39
Level 2	49.40	50.02	47.29	47.33
Level 3	49.30	49.52	48.48	49.87
Level 4	47.51	50.25	53.59	49.45
Delta	2.33	4.01	6.92	2.54
Rank	4	2	1	3

**Table 5:** Analysis of Variance (ANOVA) results.

Source	DF (Degree of Freedom)	Adj SS (Adjusted Sum of Squares)	Adj MS (Adjusted Mean Squares)	F-Value	*p*-Value	Cont. Ratio %
Printing Temperature	3	31,670	10,557	0.74	0.596	7.17
Infill Pattern	3	66,849	22,283	1.55	0.363	15.12
Infill Density	3	265,244	88,415	6.16	0.085	60.01
Layer Height	3	35,208	11,736	0.82	0.564	7.97
Error	3	43,027	14,342			9.73
Total	15	441,997				100

## Data Availability

Datasets related to these studies, findings, and results as reported are included in the manuscript itself.

## Abbreviations

The following abbreviations are used in this manuscript:

SLM	Selective Laser Melting
PBF	Powder Bed Fusion
SLA	Stereolithography
DED	Direct Energy Deposition
MEX	Material Extrusion
CONT	Contribution
DF	Degree of freedom
Adj SS	Adjusted sum of squares
Adj MS	Adjusted mean squares
SNR	Signal-to-noise ratio
ABS	Acrylonitrile Butadiene Styrene
PLA	Polylactic Acid
TPU	Thermoplastic Polyurethane
PET-G	Polyethylene Terephthalate Glycol
ANOVA	Analysis of Variance
CV	Coefficient of variation
